# Supplementary material for: Population attributable fractions of cancer mortality related to indoor air pollution, animal contact, and water source as environmental risk factors: Findings from the Golestan Cohort Study
Source: PLoS One. 2024 Jun 10;19(6):e0304828. doi: 10.1371/journal.pone.0304828 (PMC11164345; doi:10.1371/journal.pone.0304828)
Supplement: Attachment — Submitted filename: Reviewer feedback.docx [file pone.0304828.s001.docx]

**Title:**

**Population Attributable Fractions of Cancer Mortality Related to Environmental Risk Factors: The Golestan Cohort Study**

Since cancer is the major health challenge globally, i would like to thank the authors for drafting this manuscript. The manuscript is well organized and scientifically sounds for publication. Please find my concerns below.

**Concern one on title:** The phrase “environmental factors” are more general as compared to environmental risk factors attributable to cancer mortality included in this study. It is very difficult to include all environmental risk factors attributable to cancer mortality in this study. Hence, this needs to be justified and/or modification of the title.

**Concern two: Abstract discussion Section:** It is not recommended to add discussion in the **abstract section. Instead it is highly advisable to include conclusion and highlight of the** recommendation section about what to do to reduce cancer mortality as a result of exposure to various environmental risk factors.

**Concern Three: introduction section: Line 59-60:** *“Worldwide, it is estimated 60 that around 20% of premature cancer deaths are due to environmental risk factors”* If this is known, what is the unknown here? Please provide your justification.

**Concern: four Introduction section**: Is there similar articles published before? Or is it the only study on the issue? If there is previous similar work, what was the gap the authors identified and wants to fill, so that the international community can gain new insights?

**Concern Five:** How cofounders can practically be controlled. A cancer patient may be died during follow up period because of other co-morbidities.
